# Endometrial HOXA-10, HOXA-11, ß-1 integrin, ECM-1, FAK, and CD44 immunohistochemical expressions in endometriosis-related recurrent IVF failure: a retrospective case-control study

**DOI:** 10.1186/s13048-026-02072-3

**Published:** 2026-04-20

**Authors:** Esin Şahin Toruk, Elif Acar Kaplan, Gülistan Sanem Sarıbaş, Özlem Erdem, Ahmet Erdem, Mehmet Erdem

**Affiliations:** 1 Department of Gynecology and Obstetrics, Faculty of Medicine, Gazi University, Ankara, Türkiye; 2 Department of Pathology, Faculty of Medicine, Gazi University, Ankara, Türkiye; 3 Department of Histology and Embryology, Gulhane Faculty of Medicine, University of Health Sciences, Ankara, Türkiye

**Keywords:** Endometriosis, Infertility, In vitro fertilization (IVF) failure, Endometrial receptivity, CD44, FAK, ECM-1, Immunohistochemistry

## Abstract

**Background:**

Endometriosis is a common cause of infertility and is frequently associated with recurrent implantation failure in assisted reproductive technologies. Impaired endometrial receptivity, mediated by altered transcription factors, adhesion molecules, and extracellular matrix components, has been proposed as a contributing mechanism. This study aimed to evaluate compartment-specific immunohistochemical expression patterns of HOXA-10, HOXA-11, CD44, β1 integrin, ECM-1, and focal adhesion kinase (FAK) in women with endometriosis-related implantation failure.

**Methods:**

This retrospective case–control study was conducted at the IVF Unit of Gazi University Faculty of Medicine. The study group consisted of 34 infertile women with surgically confirmed endometriosis and recurrent IVF failure, subdivided into pre-receptive and receptive phases based on histological dating, while fertile women without infertility or endometriosis served as controls. Endometrial biopsies were obtained during the implantation window. Immunohistochemical expression was evaluated using semi-quantitative compartment-specific scoring, with ImageJ-based analysis used as supportive. Appropriate non-parametric statistical analyses were applied, and *p* < 0.05 was considered statistically significant.

**Results:**

No statistically significant intergroup differences were observed for stromal HOXA-10 or HOXA-11 expression between control, pre-receptive, and receptive groups. Glandular CD44 positivity was significantly increased in the receptive group compared with controls, while no significant difference was detected between pre-receptive and receptive phases after multiple comparison correction. β1 integrin expression did not demonstrate consistent phase-specific differences. In contrast, strong glandular ECM-1 expression was significantly reduced in both pre-receptive and receptive groups compared with controls, whereas stromal ECM-1 expression remained unchanged. Stromal FAK expression was significantly increased in both pre-receptive and receptive groups relative to controls, with no significant difference between these phases.

**Conclusion:**

Endometriosis-related implantation failure is associated with distinct, compartment-specific alterations in endometrial receptivity markers that appear largely independent of physiological implantation timing. Reduced glandular ECM-1 expression and persistent stromal FAK accumulation suggest disease-specific epithelial–stromal dysregulation rather than delayed or shifted receptivity. Altered glandular CD44 expression in the receptive phase likely reflects endometriosis-associated epithelial adhesion changes rather than a phase-specific marker of functional receptivity. These findings highlight the importance of compartment-focused evaluation of endometrial receptivity in endometriosis-related infertility.

**Trial registration:**

This study was retrospectively conducted and was not registered in a clinical trial registry.

## Introduction

Successful implantation requires that a competent blastocyst encounters a receptive endometrium during the window of implantation [1]. Current research seeks to clarify determinants of endometrial receptivity through genetic, biochemical, immunologic, and histologic approaches. Numerous studies indicate impaired receptivity in women with endometriosis, consistent with its adverse effect on fertility [2, 3].

Endometriosis is an estrogen-dependent condition that also exhibits progesterone resistance, which may dysregulate endometrial maturation and lead to implantation failure [4]. Although early oocyte-donation studies suggested that infertility in endometriosis might primarily reflect oocyte quality [5], more recent, larger studies implicate defects in implantation as well [6]. In women with unilateral endometriomas, embryo quality may be comparable to tubal-factor infertility; however, implantation rates are decreased and ovarian response to stimulation may be delayed [7].

Among putative receptivity markers, the Homeobox transcription factors HOXA10 and HOXA11 are expressed in the endometrium and participate in the development of pinopodes, structures implicated in conceptus–endometrium adhesion [8]. Reduced expression of these genes has been reported in several benign gynecologic disorders—including endometriosis, hydrosalpinx, and adenomyosis—and has been associated with implantation failure [9]. CD44, a hyaluronan receptor involved in cell migration, T-cell activation, and intercellular communication-cell activation, and intercellular communication, serving as a receptor for hyaluronic acid [10]. Studies have shown reduced endometrial CD44 expression in infertile patient groups [11]. Focal Adhesion Kinase (FAK) is a key protein expressed in focal adhesion sites, playing a significant role in integrin-mediated signal transduction [12]. Studies have shown higher FAK expression physiologically in the decidua [13]. Beta-1 Integrin (β1 Integrin), a surface protein from the integrin family, plays a crucial role in cell migration, intercellular communication, and tissue repair [14]. Similar to its critical role in cell adhesion, increased expression of β1 Integrin in the endometrium and decidua during early pregnancy and implantation has been demonstrated [13]. Extracellular Matrix Protein 1 (ECM1) is a secreted extracellular matrix glycoprotein [15]. Limited studies indicate reduced ECM1 expression in the endometrium of infertile patients [11].

This study aims to compare the immunohistochemical (IHC) expressions of HOXA-10, HOXA-11, CD44, β1 integrin, ECM1, and FAK in biopsies obtained during the implantation period from patients with infertility due to endometriosis who underwent unsuccessful IVF with those obtained from fertile women.

## Materials and methods

### Study design and patient selection

This retrospective case–control study was conducted at the IVF Unit of Gazi University Faculty of Medicine Hospital. The study population consisted of women aged 20–40 years with regular menstrual cycles, no endocrinological disorders, a confirmed diagnosis of endometriosis-related infertility, and a history of recurrent IVF failure. All patients underwent endometrial biopsy planned during the implantation window (cycle days 21–24).

Endometrial biopsy timing was determined using two clinically accepted approaches. In eligible patients, daily transvaginal ultrasonography and serum luteinizing hormone (LH) monitoring were initiated from cycle day 11. Following detection of the LH surge and confirmation of progesterone elevation on the subsequent day, endometrial biopsy was performed at LH + 6/7. In patients for whom intensive hormonal monitoring was not feasible due to clinical or logistical reasons, biopsy timing was scheduled according to the last menstrual period (cycle days 21–24).

Despite clinically appropriate biopsy timing within the implantation window, histopathological evaluation revealed that 13 patients demonstrated endometrial dating corresponding to cycle days 17–19 according to Noyes’ criteria. These cases were classified as exhibiting histological endometrial asynchrony (out-of-phase endometrial dating) and were defined as a distinct subgroup within the study cohort, referred to as the pre-receptive group. This finding was not considered a consequence of biopsy timing error but was interpreted as reflecting a potential biological alteration associated with endometriosis. The pre-receptive group represents a subgroup of the primary study population and was analyzed separately to evaluate the possible association between histologically identified endometrial asynchrony and implantation failure.

The control group consisted of women aged 20–40 years who presented to the same institution during the same study period, had no history of infertility or endometriosis, and no use of medications affecting the endometrium. These patients underwent endometrial biopsy for gynecological indications other than abnormal uterine bleeding, and without suspected endometrial pathology, and histopathological examination incidentally demonstrated endometrial dating consistent with the implantation window (cycle days 21–24 based on the last menstrual period).

Exclusion criteria included failure to meet inclusion criteria, infertility due to causes other than endometriosis, use of medications affecting endometrial receptivity, presence of systemic disease or estrogen/progesterone receptor–related malignancy, and biopsy findings of endometrial malignancy, hyperplasia, polyps, or chronic endometritis.

### Endometrial biopsy procedure

Endometrial biopsies were obtained under sterile conditions following appropriate field preparation, using either general or local anesthesia. All biopsies were performed in the lithotomy position using a Pipelle catheter (Pipelle^®^, Prodimed, France), a flexible polypropylene endometrial biopsy device with a diameter of less than 3 mm.

### Morphological evaluation

Histopathological evaluation was performed on archived endometrial specimens fixed in 10% neutral buffered formalin, processed routinely, embedded in paraffin, and stained with hematoxylin and eosin (H&E). Tissue sections were examined using a light microscope (Olympus BX50) at magnifications of ×12.5, ×40, ×100, ×200, and ×400.

H&E-stained sections were re-evaluated for glandular mitotic activity, nuclear pseudostratification, basal cytoplasmic vacuolization, stromal edema, pseudodecidual reaction, stromal mitosis, and leukocyte infiltration. Endometrial dating was determined according to Noyes’ criteria.

### Immunohistochemical analysis

Immunohistochemical staining was performed on selected paraffin blocks to evaluate the expression of HOXA-10, HOXA-11, β1 integrin, FAK, CD44, and ECM1 using the streptavidin–biotin triple indirect immunoperoxidase method. Antibodies and positive controls were as follows: Anti-HOXA-10 (goat polyclonal, 1:100, Abcam, USA; positive control: testis), Anti-HOXA-11 (rabbit polyclonal, 1:500, ThermoFisher Scientific, USA; positive control: endometrium), Anti-β1 integrin (rabbit monoclonal, clone EPR16895, 1:1000, Abcam, USA; positive control: colon), Anti-FAK (rabbit monoclonal, clone EP695Y, 1:250, Abcam, USA; positive control: spleen and hepatocellular carcinoma), Anti-CD44 (rabbit monoclonal, clone EPR1013Y, 1:100, Abcam, USA; positive control: tonsil), and Anti-ECM1 (rabbit monoclonal, clone EPR6701, 1:250, Abcam, USA; positive control: kidney).

Negative controls were included in all immunohistochemical staining runs by applying the same staining protocol without the primary antibody.

### Evaluation of immunohistochemical staining

Membranous staining of β1 integrin and cytoplasmic staining of CD44, FAK, and ECM1 were evaluated separately in endometrial glands and stroma. For HOXA-10 and HOXA-11, only stromal nuclear staining was assessed, as glandular nuclear expression was absent. For membranous staining, the intercellular borders of glandular epithelial cells were considered.

Staining observed in less than 10% of cells or complete absence of staining was classified as negative, whereas staining in 10–100% of cells was considered positive. Staining intensity was categorized as mild or strong by comparison with positive control tissues. Glandular and stromal staining intensities were recorded separately. Initial intergroup comparisons were performed based on the presence or absence of staining; cases without statistically significant differences were subsequently re-evaluated according to staining intensity.

Immunohistochemical evaluation was performed by a single experienced pathologist who was blinded to all clinical and histopathological group assignments. Representative, non-overlapping glandular and stromal areas were selected for evaluation, and no clinical information was available to the observer during assessment.

To support semi-quantitative scoring, digital image analysis was performed on selected samples under standardized conditions using ImageJ software. For this analysis, four samples per group were evaluated, and six representative fields per sample were analyzed at ×200 magnification. The number of samples included in ImageJ analysis was intentionally limited to ensure uniform staining quality and identical imaging conditions across all groups.

### Statistical evaluation

Statistical analyses were performed using SPSS for Windows, version 22.0 (Statistical Package for the Social Sciences, IBM Corp., Armonk, NY, USA). The normality of data distribution was assessed using the Kolmogorov–Smirnov and Shapiro–Wilk tests. Continuous variables are presented as mean ± standard deviation, while categorical variables are expressed as numbers and percentages. Comparisons of categorical variables between groups were performed using the Chi-square test. For comparisons of three independent groups involving continuous variables that did not follow a normal distribution, the Kruskal–Wallis test was applied. Associations between continuous variables were evaluated using Spearman’s rank correlation coefficients. All statistical analyses were conducted using a 95% confidence level, and a p-value < 0.05 was considered statistically significant. Given the retrospective study design and the inability of ImageJ-based analysis to reliably distinguish between glandular and stromal compartments, ImageJ-derived continuous variables were not used as primary statistical endpoints. Semi-quantitative immunohistochemical evaluation served as the primary analytical approach. ImageJ-based quantitative measurements were performed under standardized imaging conditions and used solely as supportive analyses.

Based on a review of the existing literature, a post-hoc power analysis was conducted using effect size estimates derived from a previously published study reporting a significant positive correlation between focal adhesion kinase (FAK) expression and endometriosis disease severity (*r* = 0.437 and *r* = 0.440, respectively; *p* = 0.022). Power calculations were performed using G*Power software version 3.1 (Heinrich Heine University, Düsseldorf, Germany). Assuming a two-tailed test with an alpha level of 0.05 and a statistical power of 80%, the minimum required total sample size was calculated as 47 patients. Given that the present study included 55 patients, the sample size was considered sufficient for the planned analyses.

## Results

### Demographic and clinical characteristics

A total of 55 patients were included in the study and categorized into three groups: control (*n* = 21), pre-receptive (*n* = 13), and receptive (*n* = 21). A statistically significant difference in age was observed among the groups (*p* = 0.018, Table 1). Pairwise comparisons indicated that this difference was primarily driven by the comparison between the control and pre-receptive groups, whereas no significant difference was detected between the pre-receptive and receptive groups. Body mass index (BMI) also differed significantly among the groups (*p* < 0.001, Table 1). The control group exhibited significantly higher BMI values compared with both the pre-receptive and receptive groups. No statistically significant difference was observed between the pre-receptive and receptive groups.

**Table 1:** Comparison of age and body mass index among control, pre-receptive, and receptive groups

	Control Group	Pre-receptive Group	Receptive Group	
Variable	Mean ± SD	Mean ± SD	Mean ± SD	*p* value
Age	36.67 ± 2.76	33.31 ± 3.64	34.57 ± 3.19	0.018*
Body Mass Index (kg/m²)	25.07 ± 3.54	21.10 ± 1.37	21.69 ± 1.18	0.000*

Statistical test: Kruskal–Wallis test

* Statistical significance was defined as *p* < 0.05

Significant intergroup differences were identified with respect to gravida, parity, and number of live births (Table 2). These parameters were significantly higher in the control group compared with both the pre-receptive and receptive groups. In contrast, no statistically significant differences were observed among the groups regarding the number of abortions, biochemical pregnancies, or ectopic pregnancies.

**Table 2:** Comparison of gravidity, parity, pregnancy outcomes, and live births among control, pre-receptive, and receptive groups

	Control Group (*n* = 21)	Pre-receptive Group (*n* = 13)	Receptive Group (*n* = 21)	
Variable	Mean ± SD	Mean ± SD	Mean ± SD	*p* value
Gravidity	2.05 ± 0.92	1.08 ± 1.04	0.86 ± 0.85	0.000*
Parity	1.81 ± 0.87	0.31 ± 0.48	0.14 ± 0.48	0.000*
Abortions	0.19 ± 0.40	0.69 ± 1.18	0.52 ± 0.60	0.134
Biochemical pregnancy	0.00 ± 0.00	0.08 ± 0.28	0.14 ± 0.36	0.210
Ectopic pregnancy	0.00 ± 0.00	0.00 ± 0.00	0.05 ± 0.22	0.445
Live births	1.57 ± 0.81	0.23 ± 0.44	0.05 ± 0.22	0.000*

Statistical test: Kruskal–Wallis test

* Statistical significance was defined as *p* < 0.05

Comparisons between the pre-receptive and receptive groups revealed no statistically significant differences in infertility duration, number of ART attempts, antral follicle count (AFC), or number of retrieved oocytes (Table 3)

**Table 3:** Comparison of infertility duration, number of ART attempts, antral follicle count, and number of retrieved oocytes between pre-receptive and receptive groups

	Pre-receptive Group (*n* = 13)	Receptive Group (*n* = 21)	
Variable	Mean ± SD	Mean ± SD	*p* value
Infertility duration (years)	12.77 ± 5.88	9.43 ± 4.51	0.066
Number of ART attempts	3.15 ± 1.41	3.86 ± 1.71	0.180
Antral follicle count (AFC)	12.77 ± 5.88	9.43 ± 4.51	0.066
Number of retrieved oocytes	14.69 ± 6.26	10.90 ± 6.28	0.096

Mann–Whitney U test was used for all comparisons

### Glandular immunohistochemical staining findings

Glandular immunohistochemical staining patterns of ECM1, β1 integrin, CD44, and FAK were evaluated (Table 4).

**Table 4:** Glandular immunohistochemical staining distribution of ECM1, β1 integrin, CD44, and FAK among study groups

Marker	Staining status	Control Group (n:21)		Pre-receptive Group (*n* = 13)		Receptive Group (*n* = 21)	
		*n*	%	*n*	%	*n*	%
ECM1	Negative	9	42.86	11	84.62	17	80.95
	Positive	12	57.14	2	15.38	4	19.05
β1 Integrin	Negative	1	4.76	0	0.00	1	4.76
	Positive	20	95.24	13	100.00	20	95.24
CD44	Negative	9	42.86	5	38.46	2	9.52
	Positive	12	57.14	8	61.54	19	90.48
FAK	Negative	21	100.00	12	92.31	21	100.00
	Positive	0	0.00	1	7.69	0	0.00

A statistically significant difference was observed among the groups regarding the presence of glandular ECM1 staining (*p* = 0.010, Table 5). Pairwise comparisons demonstrated that this difference was mainly attributable to the comparison between the control and receptive groups, whereas no significant difference was observed between the pre-receptive group and the other groups. Further sub-analysis based on staining intensity revealed that strong glandular ECM1 staining was significantly more frequent in the control group compared with both the pre-receptive and receptive groups (*p* = 0.004, Table 5).

**Table 5:** Comparison of glandular ECM1 staining presence and intensity among control, pre-receptive, and receptive groups

		Control Group (n:21)		Pre-receptive Group (*n* = 13)		Receptive Group (*n* = 21)		
		*n*	%	*n*	%	*n*	%	*p* value
Presence of glandular ECM1 staining	Negative	12	57.14	2	15.38	4	19.05	0.010*
	Positive	9	42.86	11	84.62	17	80.95	
Weak glandular ECM1 staining	Negative	15	71.43	11	84.62	17	80.95	0.615
	Positive	6	28.57	2	15.38	4	19.05	
Strong glandular ECM1 staining	Negative	15	71.43	13	100.00	21	100.00	0.004*
	Positive	6	28.57	0	0.00	0	0.00	

* Statistical significance was defined as *p* < 0.05; Bonferroni correction was applied for post-hoc pairwise comparisons; adjusted significance level was set at *p* < 0.017. Post-hoc results for glandular ECM1 staining presence: Control vs. Pre-receptive: *p* = 0.018; Control vs. Receptive: *p* = 0.012; Pre-receptive vs. Receptive: *p* = 0.789. Post-hoc results for strong glandular ECM1 staining: Control vs. Pre-receptive: *p* = 0.036; Control vs. Receptive: *p* = 0.009

For CD44, the presence of glandular staining differed significantly among the groups (p = 0.041, Table 6). This difference was primarily driven by the comparison between the control and receptive groups. However, no statistically significant differences were observed when glandular CD44 staining was analyzed according to staining intensity

**Table 6:** Comparison of glandular CD44 staining presence and intensity among control, pre-receptive, and receptive groups

		Control Group (n:21)		Pre-receptive Group (*n* = 13)		Receptive Group (*n* = 21)		
		*n*	%	*n*	%	*n*	%	*p* value
Presence of glandular CD44 staining	Negative	12	57	8	62	19	90	0.041*
	Positive	9	43	5	38	2	10	
Weak glandular CD44 staining	Negative	16	76	10	77	15	71	0.916
	Positive	5	24	3	23	6	29	
Strong glandular CD44 staining	Negative	14	67	8	62	8	38	0.150
	Positive	7	33	5	38	13	62	

* Statistical significance was defined as *p* < 0.05; Bonferroni correction was applied for post-hoc pairwise comparisons; the adjusted significance threshold was set at *p* < 0.017. Post-hoc analysis for glandular CD44 staining presence: Control vs. Pre-receptive: *p* = 0.803; Control vs. Receptive: *p* = 0.015; Pre-receptive vs. Receptive: *p* = 0.046

No statistically significant intergroup differences were detected with respect to glandular β1 integrin or FAK staining, either in terms of staining presence or staining intensity.

**Fig. 1:**
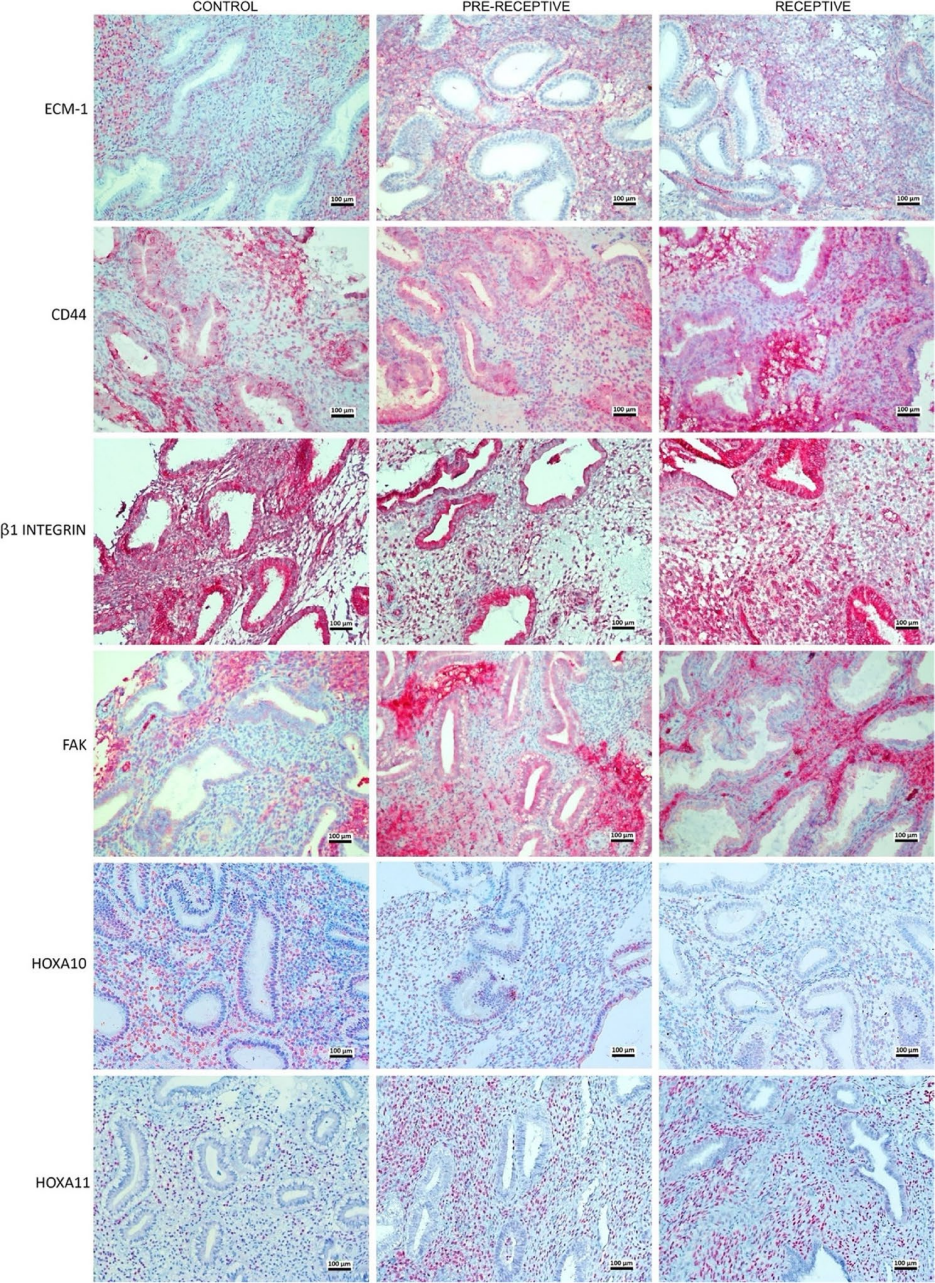
Representative immunohistochemical staining patterns of ECM1, CD44, β1 integrin, FAK, HOXA10, and HOXA11 in control, pre-receptive, and receptive endometrial samples. Original magnification ×200; AEC chromogen with hematoxylin counterstaining

Representative immunohistochemical staining patterns for ECM1, CD44, β1 integrin, FAK, HOXA10, and HOXA11 across the control, pre-receptive, and receptive groups are shown in Fig. 1.

### Stromal immunohistochemical staining findings

Stromal immunohistochemical staining patterns of ECM1, β1 integrin, CD44, FAK, HOXA10, and HOXA11 were evaluated (Table 7).

**Table 7:** Distribution of stromal immunohistochemical staining among control, pre-receptive, and receptive groups

Marker	Staining status	Control Group (n:21)		Pre-receptive Group (*n* = 13)		Receptive Group (*n* = 21)	
		*n*	%	*n*	%	*n*	%
ECM1	Negative	4	19.05	0	0.00	3	14.29
	Positive	17	80.95	13	100.00	18	85.71
β1 Integrin	Negative	9	42.86	8	61.54	8	38.10
	Positive	11	57.14	3	38.46	13	61.90
CD44	Negative	4	19.05	2	15.38	1	4.76
	Positive	17	80.95	11	84.62	20	95.24
FAK	Negative	2	9.52	0	0.00	0	0.00
	Positive	19	90.48	13	100.00	21	100.00
HOXA10	Negative	4	19.05	6	46.15	9	42.86
	Positive	17	80.95	7	53.85	12	57.14
HOXA11	Negative	0	0.00	0	0.00	0	0.00
	Positive	21	100.00	13	100.00	21	100.00

No statistically significant differences were observed among the control, pre-receptive, and receptive groups regarding stromal ECM1, β1 integrin, or CD44 staining, either in terms of presence or staining intensity.

Although no significant intergroup difference was found in the presence of stromal FAK staining, a statistically significant difference was observed with respect to stromal FAK staining intensity (*p* < 0.001, Table 8). Strong stromal FAK staining was significantly more frequent in both the pre-receptive and receptive groups compared with the control group. Pairwise comparisons indicated that this difference was driven by comparisons between the control group and each of the other two groups, whereas no significant difference was observed between the pre-receptive and receptive groups.

**Table 8:** Comparison of stromal FAK staining presence and intensity among control, pre-receptive, and receptive groups

		Control Group (n:21)		Pre-receptive Group (*n* = 13)		Receptive Group (*n* = 21)		
		*n*	%	*n*	%	*n*	%	*p* value
Stromal FAK staining	Negative	19	0.90	13	1.00	21	1.00	0.186
	Positive	2	0.10	0	0.00	0	0.00	
Weak stromal FAK staining	Negative	11	0.52	12	0.92	21	1.00	0.000*
	Positive	10	0.48	1	0.08	0	0.00	
Strong stromal FAK staining	Negative	12	0.57	1	0.08	0	0.00	0.000*
	Positive	9	0.43	12	0.92	21	1.00	

*p < 0,05; Bonferroni correction was applied for post-hoc pairwise comparisons; adjusted significance threshold was set at *p* < 0.017. Weak stromal FAK staining (post-hoc): Control vs. Pre-receptive: *p* = 0.017; Control vs. Receptive: *p* < 0.001; Pre-receptive vs. Receptive: *p* = 0.204. Strong stromal FAK staining (post-hoc): Control vs. Pre-receptive: *p* = 0.005; Control vs. Receptive: *p* < 0.001; Pre-receptive vs. Receptive: *p* = 0.204

No statistically significant differences were detected among the groups regarding stromal HOXA10 or HOXA11 expression, either in terms of staining presence or staining intensity.

## Discussion

The human endometrium is a critical determinant of fertility [1]. Numerous studies have investigated genetic, biochemical, immunological, and histological determinants of endometrial receptivity; however, the precise molecular mechanisms governing successful implantation remain incompletely understood. Despite the pivotal role of the window of implantation, histological dating alone demonstrates only a weak correlation with fertility outcomes [16–20].

Although early oocyte donation studies suggested that infertility in endometriosis predominantly reflects impaired oocyte quality [5], accumulating evidence indicates that endometrial receptivity is also substantially altered in this population [6]. Defective decidualization has been consistently demonstrated in women with endometriosis [2, 3], emphasizing the contribution of uterine factors. While transcriptomic tools such as the Endometrial Receptivity Array have questioned the clinical utility of single-gene assessments [21], recent data increasingly support functional and compartment-specific alterations within the endometrial microenvironment in endometriosis [22, 23].

In this context, the present study provides a comprehensive immunohistochemical evaluation of key adhesion- and receptivity-related markers in endometrial biopsies obtained during the implantation window from women with endometriosis-related recurrent implantation failure, with a particular focus on compartment-specific (glandular versus stromal) alterations. 

In contrast to some earlier reports, our study did not demonstrate statistically significant intergroup differences in stromal HOXA10 or HOXA11 immunohistochemical expression among control, pre-receptive, and receptive groups. HOXA transcription factors are known to play a central role in implantation by regulating cell–cell and cell–extracellular matrix adhesion. Their expression is progesterone-dependent and closely linked to pinopode formation and integrin regulation during the window of implantation [8]. Previous studies have reported reduced HOXA10 and HOXA11 expression in women with endometriosis, with partial restoration following surgical treatment [9]. However, discrepancies between mRNA-level changes and protein-level immunohistochemical findings have also been documented [24]. The absence of detectable differences in HOXA10 and HOXA11 protein expression in our cohort may reflect differences between transcript and protein levels and/or methodological sensitivity limitations of immunohistochemistry, rather than true preservation of functional HOXA signaling. These findings suggest that HOXA dysregulation in endometriosis may not be uniformly reflected at the protein level during the implantation window.

CD44 is a transmembrane glycoprotein that serves as a principal receptor for hyaluronic acid and plays a key role in embryo–endometrium interaction and vascular invasion [18]. Previous studies have shown that CD44 expression increases during the secretory phase of the endometrium, coinciding with the window of implantation [18, 19]. Moreover, aberrant upregulation of CD44 has been implicated in the excessive invasiveness characteristic of endometriosis pathophysiology [10]. In the present study, glandular CD44 positivity was significantly increased in the receptive group compared with the control group. In contrast, no significant difference was observed between the pre-receptive and receptive phases after Bonferroni correction.

Consistent with previous reports indicating that aberrant upregulation of CD44 in endometriosis is associated with an excessively invasive epithelial phenotype [10], the increased glandular CD44 positivity observed in the receptive group in our cohort may reflect disease-specific alterations in epithelial adhesion rather than serving as a precise marker of implantation timing.

Integrins are large transmembrane proteins that mediate cell–cell and cell–extracellular matrix adhesion during implantation. Although β1 integrin has been proposed as a marker of endometrial receptivity, its expression spans multiple phases of the menstrual cycle [14]. In our study, no statistically significant differences were detected among the groups with respect to glandular or stromal β1 integrin expression. This finding aligns with previous observations indicating that β1 integrin lacks sufficient phase specificity to reliably define the implantation window [14]. Our results further support the notion that β1 integrin expression alone is insufficient to discriminate functional endometrial receptivity in women with endometriosis-related implantation failure.

Following integrin engagement, focal adhesion kinase (FAK) is activated and orchestrates cytoskeletal remodeling, cellular adhesion dynamics, and tissue permeability [20]. In the present study, glandular FAK staining was minimal and did not differ significantly among the groups. In contrast, stromal FAK staining intensity demonstrated marked and statistically significant intergroup differences, with both weak and strong stromal FAK staining being significantly more prevalent in pre-receptive and receptive groups compared with controls. The absence of a significant difference between the pre-receptive and receptive groups suggests that increased stromal FAK accumulation is not dynamically regulated by physiological implantation timing, but rather represents a persistent endometriosis-associated stromal alteration. This persistence across phases indicates that histological asynchrony and enhanced stromal FAK signaling may be consistent with an intrinsic feature of endometriosis-related endometrial dysfunction, potentially interfering with receptivity-related remodeling and adversely affecting implantation. Previous studies have demonstrated increased FAK expression in endometrial tissues of women with endometriosis and have linked FAK activation to disease severity, pelvic pain, and steroid hormone milieu [12]. Experimental models further suggest that excessive focal adhesion formation may increase stromal rigidity, thereby acting as a biomechanical barrier to embryo invasion [25]. Our findings support the hypothesis that increased stromal FAK accumulation, rather than facilitating embryo penetration, may reinforce a mechanical and adhesive barrier within the endometrial stroma and suggest that this alteration may adversely affect implantation.

Extracellular matrix proteins play a crucial role in early embryo adhesion and stromal remodeling. ECM-1 is a secreted glycoprotein that has been shown to increase at the maternal–fetal interface during early pregnancy [15]. Conversely, reduced ECM-1 levels have been reported in infertile women [11]. In the present study, glandular ECM-1 expression—particularly strong glandular staining—was significantly reduced in both pre-receptive and receptive groups compared with controls, whereas stromal ECM-1 expression did not differ significantly among the groups. Notably, the reduction in glandular ECM-1 expression was most pronounced when comparing the control and receptive groups, while no significant difference was observed between the pre-receptive and receptive phases. This pattern suggests that ECM-1 dysregulation is not merely related to endometrial timing, but rather reflects a disease-specific impairment of epithelial function during the window of implantation. The selective loss of strong glandular ECM-1 staining in the receptive phase underscores its potential role in defective epithelial–stromal crosstalk contributing to implantation failure in endometriosis.

### Overall interpretation

Taken together, our findings demonstrate that endometriosis-related implantation failure is characterized by distinct, compartment-specific alterations in endometrial receptivity markers. Rather than reflecting a global loss of receptivity, the observed pattern—reduced glandular ECM-1 expression, altered glandular CD44 presence, and pronounced stromal FAK accumulation—suggests maladaptive remodeling of the endometrial microenvironment.

These data support the concept that implantation failure in endometriosis is driven by disease-specific epithelial–stromal dysregulation rather than a simple shift or delay in endometrial timing. This compartment-focused perspective provides a more nuanced understanding of implantation failure in endometriosis and underscores the importance of stromal–epithelial balance rather than single-marker evaluation. Although the sample size was statistically adequate based on post-hoc power analysis, these findings should nevertheless be interpreted with caution due to the retrospective design and the limited size of specific subgroups. Larger prospective studies are warranted to confirm and extend these associations.

Importantly, the absence of significant differences between the pre-receptive and receptive groups across several markers suggests that the observed alterations are not primarily driven by physiological implantation timing. Rather, this pattern indicates that these molecular changes likely reflect disease-related endometrial dysfunction associated with endometriosis itself. The persistence of altered glandular and stromal expression profiles across both phases supports the concept that endometriosis disrupts the endometrial microenvironment in a phase-independent manner, potentially predisposing the endometrium to implantation failure despite clinically appropriate timing.

### Limitations and considerations

While the present study provides novel insights into compartment-specific alterations of endometrial receptivity markers in women with endometriosis-related implantation failure, several limitations should be acknowledged. First, the overall sample size was relatively small, particularly in the pre-receptive subgroup, which may have limited the statistical power to detect subtle intergroup differences. Although post-hoc power analysis indicated adequate power for the primary comparisons, the findings should nevertheless be interpreted with caution. ImageJ-based quantitative analyses demonstrated trends largely consistent with the semi-quantitative immunohistochemical findings, particularly for stromal FAK accumulation and glandular ECM-1 alterations. However, because robust and reproducible compartment-specific segmentation (glandular versus stromal) could not be ensured across retrospectively archived samples, ImageJ-derived quantitative results were interpreted as supportive rather than definitive. Accordingly, semi-quantitative, compartment-specific immunohistochemical evaluation served as the primary analytical approach in this study.

Second, the retrospective case–control design precludes any causal inference between altered marker expression and implantation failure. The observed associations reflect disease-related molecular patterns rather than direct mechanistic effects. In addition, potential confounding factors such as endometriosis stage, duration of infertility, and IVF-related parameters could not be fully controlled for, which may have influenced the observed expression profiles.

Third, immunohistochemistry is a semi-quantitative technique and is inherently subject to technical variability and observer-dependent interpretation. Although standardized staining protocols were applied, subtle differences in protein expression may not be fully captured at the immunohistochemical level. Moreover, the lack of parallel molecular validation methods (e.g., gene expression or protein quantification assays) represents an additional limitation.

Finally, the classification of pre-receptive and receptive groups was based on histological dating, which itself has known limitations in precisely defining functional endometrial receptivity. Therefore, the absence of significant differences between these phases for several markers should be interpreted in the context of these methodological constraints.

### Future directions

Future studies should aim to validate these findings in larger, prospectively designed cohorts with balanced group sizes and well-matched controls. Multicenter studies incorporating comprehensive clinical, hormonal, and IVF-related variables would allow for more robust adjustment of potential confounders and improve generalizability.

Importantly, combining compartment-specific immunohistochemical evaluation with advanced digital image analysis methods capable of reliable glandular–stromal segmentation, as well as molecular and functional assays, may provide deeper insight into the biological significance of the observed alterations.

Longitudinal sampling across the menstrual cycle could further clarify whether these molecular patterns represent stable disease-related traits or dynamic changes modulated by hormonal signaling. Given the prominent alterations observed in stromal FAK and glandular ECM-1 expression, future research should particularly focus on epithelial–stromal interactions, extracellular matrix remodeling, and endometrial biomechanics in endometriosis. Such integrative approaches may help bridge the gap between descriptive histopathological findings and functional mechanisms underlying implantation failure.

## Conclusions

Endometriosis-related implantation failure is associated with distinct, compartment-specific alterations in endometrial receptivity. Reduced glandular ECM-1 expression and increased stromal FAK accumulation were consistently observed across both pre-receptive and receptive phases, suggesting persistent epithelial–stromal dysregulation rather than a simple shift in implantation timing. Increased glandular CD44 positivity in the receptive phase appears to reflect disease-related epithelial adhesion changes rather than a phase-specific receptivity marker.

These findings support the concept that endometriosis induces phase-independent endometrial dysfunction characterized by maladaptive remodeling of the epithelial–stromal interface. While associative in nature, our results highlight the importance of compartment-focused evaluation of endometrial receptivity and warrant further integrative studies to clarify the underlying mechanisms of implantation failure in endometriosis.

## Data Availability

The datasets used and/or analyzed during the current study are available from the corresponding author on reasonable request.
